# Dynamics of germination stimulants dehydrocostus lactone and costunolide in the root exudates and extracts of sunflower

**DOI:** 10.1080/15592324.2022.2025669

**Published:** 2022-01-21

**Authors:** Wenlong Wu, Hongjuan Huang, Jietian Su, Xiaopeng Yun, Yixiao Zhang, Shouhui Wei, Zhaofeng Huang, Chaoxian Zhang, Quanjiang Bai

**Affiliations:** a Institute of Plant Protection Chinese Academy of Agricultural Sciences, Beijing, China; b Institute of Plant Protection, Inner Mongolia Academy of Agriculture and Animal Husbandry Sciences, Hohhot, China

**Keywords:** Sunflower, *Orobanche cumana* Loefl, dehydrocostus lactone, costunolide

## Abstract

*Orobanche cumana* Wallr. (*Orobanche cernua* Loefl.) causes severe yield losses of confectionary sunflower in China. While germination of *O. cumana* is stimulated by sesquiterpene lactones (STLs) from host sunflower (*Helianthus annuus* L.). Dehydrocostus lactone and costunolide isolated from sunflower root exudates are known as STLs to specifically induce *O. cumana* germination. Two major confectionary sunflower cultivars, SH363 (highly susceptible to *O. cumana*) and TH33 (resistant to *O. cumana*), were planted in China. However, STLs in these two sunflower cultivars has remained unknown. To identify STLs from root and exudates of sunflower for better understanding the role of stimulants in parasitic interaction of sunflower and *O. cumana*, we tested dehydrocostus lactone (DCL) and costunolide (CL) in root and root exudates of susceptible and resistant sunflower cultivars. The stimulant activity of sunflower root exudate and root extract to germination of *O. cumana* were also determined. Dehydrocostus lactone and costunolide were identified through ultra-performance liquid chromatography coupled with mass spectrometry (UPLC-MS). Both DCL and CL were found in root extracts and root exudates in the whole tested time point from two sunflower cultivars. The concentration of dehydrocostus lactone was higher than that of costunolide at the same tested growth stage of each sunflower cultivar. It was observed that higher quantity of dehydrocostus lactone in susceptible cultivar than resistant cultivar of root and root exudates at later tested developmental stages. However, the amount of CL was no significant difference between SH363 and TH33 at all tested stages. The release amount of DCL from susceptible cultivar is 3.7 folds that of resistant cultivar at 28 DAT. These findings suggested that DCL was the one of the major signal compound in these two sunflower cultivars, and lower dehydrocostus lactone might contribute to the resistance of sunflower TH33 to *O. cumana*.

## 1 Introduction

The root parasitic weed *Orobanche cumana* Loefl. is a substantial threat to sunflower (*Helianthus annuus* L.) production in major planting areas all over the world except North American.^1^^,^^2^ It was first described in China since 1956 and then listed as a quarantine weed because of its significant adverse effects on sunflower productions. Before 2005, only low to moderate *O. cumana* infestation in sunflower was reported in China.^3–6^ However, in the past 15 years, along with the expansion of sunflower production, this root parasitic weed spread all over the main sunflower growing areas and led up to 100% sunflower yield losses in heavily infested plots.^7^ In our recent field survey, *O. cumana* infested 17.5–100% and over 100 emerged parasites were found on each host plant in seriously infested fields in Xinjiang and Inner Mongolia.^8^ Currently, *O. cumana* has been a major constraint to sunflower crop in China.^7–9^ It is very difficult to control this weed using traditional methods because of the physiological connection with sunflower. There are different potentially available alternative options such as crop rotation, suicidal germination, herbicide application on herbicide-resistant sunflower (imidazole treatment) or resistant sunflower cultivars for sunflower broomrape control.^10–12^ Resistant sunflower cultivars are regarded as one of the effective approaches in *O. cumana* management. Based on our field survey, only around 2% infestation of *O. cumana* in resistant sunflower hybrids compared with the 100% infestation of susceptible hybrids in the same area.^8^ For better management of this parasitic weed using resistance sources, it is very important to understand the mechanisms driving resistance to *O. cumana* in sunflower varieties. Several biological mechanisms of the interaction between *O. cumana* and sunflower have been reported.^13^ The investigations focused on induced germination in sunflower resistant and susceptible at initial development stage of *O. cumana* were reported previously. During *O. cumana* life cycle, seed germination dependents on chemical signal released by the host roots. Several investigations on *O. cumana* germination induced by root exudate of host plant were performed. In some cases, the results indicated that susceptible genotypes induced significantly higher germination compared to resistant cultivars.^13–16^ However, in other cases, it were suggested that there were no correlation in the resistance of sunflower lines and germination of broomrape seed.^17–20^ Different results may be due to various sunflower materials and broomrape populations used in the experiments and other resistant mechanisms involved at other stage than the germination. Previous studies of germination stimulants from root exudate of sunflower led to the isolation and identification of sesquiterpene lactones (STLs) including dehydrocostus lactone (DCL), costunolide (CL), tomentosin, 8-epixanthatin, and non-sesquiterpene lactone heliolactone.^21–23^ Among these, DCL and CL were effective only on *O. cumana* and *Orobanche minor* Sm. at a very low concentration; however, heliolactone was effective on other Orobanche plants.^23^ The STLs present in leaves, stems, oil extracts, cotyledons, hypocotyls, roots, and seedling of sunflower^24,25^ act as germination stimulants and signal for chemotropic signals of the parasite’s germtube.^25,26^

Confectionery sunflower SH363, a highly susceptible to *O. cumana*, is the most popular cultivar grown in China because of the economic interest. With the continuously planting of this susceptible sunflower cultivar, *O. cumana* has been becoming one of the dominant pests in sunflower production.^7,8^ In recent years, resistant cultivar TH33 was successful for management of this weed in some seriously affected area. However, it is little known the resistant mechanism of sunflower cultivar TH33. In this study, we focus on DCL and CL associated with induced germination of *O. cumana*. To determine whether difference of these two specific germination stimulants existed between resistant and susceptible sunflower cultivars, we evaluated the dynamic of DCL and CL from root exudates and root extracts of sunflower cultivars SH363 and TH33 at the different development stages.

## 2 Materials and methods

### 2.1 Plant materials and reagents

Seeds of *O. cumana* were collected in 2017 from main confectionery planting area in Xinjiang (44°30ʹ58” N, 86°24ʹ17” E) and stored (specimen code: WR0001572) in darkness at 4°C until used. Confectionery sunflower seeds of susceptible (cultivar SH363) and resistant (cultivar TH33) hybrids to *O. cumana* were used in the experiment. All the sunflower seeds were obtained from Inner Mongolia Academy of Agriculture and Animal Husbandry Sciences. Dehydrocostus lactone (DCL, purity ≧ 98%, product of Switzerland) and costunolide (CL, purity ≧ 97%, product of China) were purchased from Sigma-Aldrich (St. Louis, MO). Acetonitrile and water used for UPLC-MS and extraction were HPLC-grade.

### 2.2 Greenhouse experiments

The experiment was conducted in greenhouse with a temperature of 25°C/20°C (day/night), photoperiod of 14 h (300 µmol m^−2^s^−1^) at the institute of plant protection Chinese Academy of Agricultural Sciences. Five plastic pots (ø 33 cm) were filled with a mixture of top soil sampled from an arable land and sand (soil: sand 3:1 by volume) to a total weight of 6.50 kg. In each pot, 0.5 g of *O. cumana* seeds was mixed evenly, and sunflower seeds were sown and covered with mixed soil. Sunflower daily management with consistent conditions was carried out after seeds germination of SH363 and the number of *O. cumana* was counted once a week until 35 days.

### 2.3 Sunflower root exudates collection and extraction

The confectionary sunflower seeds were sown in a plastic tray filled with sterile sand and incubated in greenhouse with a temperature of 25°C/20°C (day/night), photoperiod of 14 h (300 µmol m^−2^s^−1^) in June 2019 at institute of plant protection Chinese Academy of Agricultural Sciences. After 2 weeks, seedlings of sunflower were gently rinsed with distilled water and transplanted to a plastic container (50 cm × 35 cm × 14 cm) containing 10 L of Hoagland solution and incubated in greenhouse. There were eight seedlings per container and total four containers as replications. The sunflower root exudates were obtained by 5 g of activated charcoal as described previously.^23^ To collect root exudates at different growth stage of sunflower, the activated charcoal and Hoagland solution were replaced on 7, 14, 21, 28, and 35 days after transplanted (7 DAT, 14 DAT, 21 DAT, 28 DAT, 35 DAT) to hydroponic medium. The root exudates absorbed by activated charcoal were eluted with acetone (80 mL) at 4°C for 1 week and evaporated under vacuum to remove acetone. The residual aqueous solution was extracted three times with chloroform. Three chloroform extracts were combined and dried under nitrogen and dissolved with acetonitrile to 500 μL followed filtered through a 0.22 μm nylon filter and stored in a brown glass vial at 4°C. This stored extracts was regarded as crude of root exudate. Four replicates were collected at each growth stage, and each from eight sunflower plants.

### 2.4 Extraction of DCL and CL from sunflower root

For the DCL and CL quantity of sunflower at different growth stages, the roots of 7, 14, 21, 28, and 35 DAT sunflower plants were harvested and rinsed with deionized water 3–5 times, then frozen in liquid nitrogen immediately. The roots were ground under liquid nitrogen, 1 g of root powder was homogenized in 5 mL acetonitrile and vortex for 3 min, and then ultrasonic extraction for 10 min followed by centrifugation (5430 R, Eppendorf AG, GM) at 10,000 rpm for 10 min. The supernatant was collected and filtrated through a 0.22 μm nylon filter. Four replicates were prepared for each growth stage.

### 2.5 Germination bioassay

Seeds of *O. cumana* were surface sterilized with 2% sodium hypochlorite for 2 min and 75% ethanol for 3 min followed rinsed three times with distilled water. For precondition of broomrape seeds, approximately 50–100 sterilized broomrape seeds were placed on a glass fiber filter (ø 8 mm, Whatman G/A) moistened with 100 μL distilled water in Petri dishes (ø 90 mm) and incubated (RXZ-380B, JN) in darkness at 25°C for 5 days. Thereafter, glass fiber filter with *O. cumana* seeds was dried on filter paper to remove the excess water and transferred to a new Petri dish (ø 90 mm). For effect of sunflower root exudates on *O. cumana* seed, the stored crude of root exudate at 28 DAT were dried again with nitrogen, and dissolved in a small amount of acetone, then diluted in distilled water to obtain 10,000-fold (10,000×) dilution. The glass fiber filter with seeds was supplied 100 μL dilution of root exudate. The control treatment was supplied 100 μL distilled water only. Four replications each had 3 glass fiber filters with seeds for each treatment. Petri dishes were sealed with parafilm and incubated at 25°C for 1 week in dark. Germinated broomrape seeds with radicles were counted under a stereo microscope 7 days after treatment.

### 2.6 UPLC-MS analyses

Both DCL and CL in root exudates and root extraction were determined using UPLC-MS (Waters Acquity/TQD). The analysis was carried out on a ACQUITY BEH C_18_ column (2.1 mm × 100 mm, 1.7 μm), with water (0.2% formic acid) as mobile phase A and acetonitrile as mobile phase B gradient elution (0–2.0 min, 90% A→10% A; 2.0–3.5 min, 10% A; 3.5–3.6 min, 10% A→90% A; 3.6–5.0 min, 90% A) at a flow rate of 0.3 mL·min^−1^. The column temperature was maintained at 30°C and the injection volume was 5 μL. DCL and CL were separated by chromatography column, and analyzed by MS under positive ion mode with multiple reaction monitoring (MRM) mode.^27^ DCL and CL were quantified by peak area ratios of selected ion chromatograms (SICs) between those analytes and standards, and calibration curves were employed to resolve the ionization efficiency differences between analytes and corresponding standards.

## 3 Statistical analysis

Data were subjected to the analysis of variance (ANOVA). Treatments means were separated and compared using the least significant difference test (LSD) at p ≤ .05. Germination (%) of *O. cumana* was converted to percent. The content of DCL/CL from root exudates was converted to the releasing quantity of per plant per day. The DCL/CL content of root extracts is presented as nmol/g fresh root at different measuring times.

## 4 Results

### 4.1 Orobanche development kinetics

**Figure 1.**
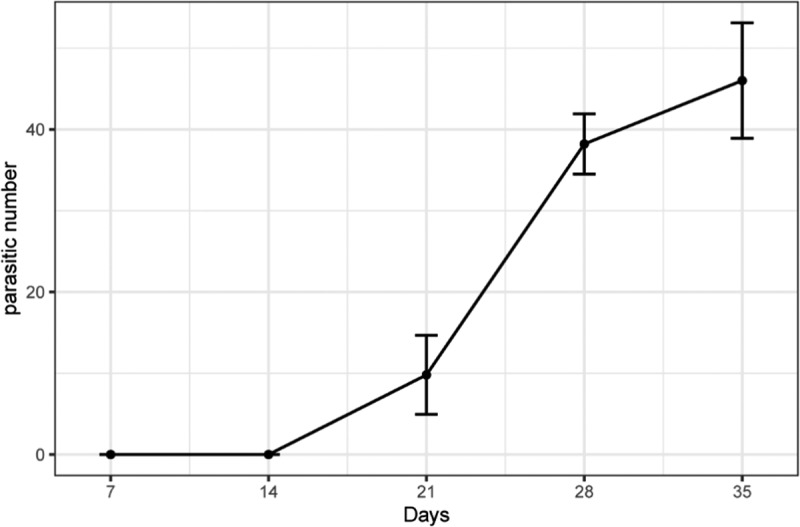
The number of *O. cumana* (*O. cernua*) on sunflower in 7–35 days.

The line graph (Figure 1) below indicates the number of *O. cumana* grew on sunflower (SH363) at five different points in time. A dramatic increase was found in the average number of *O. cumana* from 0 to 46 per sunflower plant during the entire period. By accounting for 46.0, the number of *O. cumana* at 35 DAT was the largest. The number of *O. cumana* at 28 DAT was 17% smaller than the first one, by which it took the second place. This was followed by the number of *O. cumana* at 21 DAT, with average 9.8 per sunflower plant. The number of *O. cumana* at 14, 7 DAT ranked the last, which were 0 after sowing sunflower seeds. All in all, the number of *O. cumana* saw an increase after the 14 days growth period of sunflower.

### 4.2 Quantity of DCL and CL in root exudates

**Figure 2.**
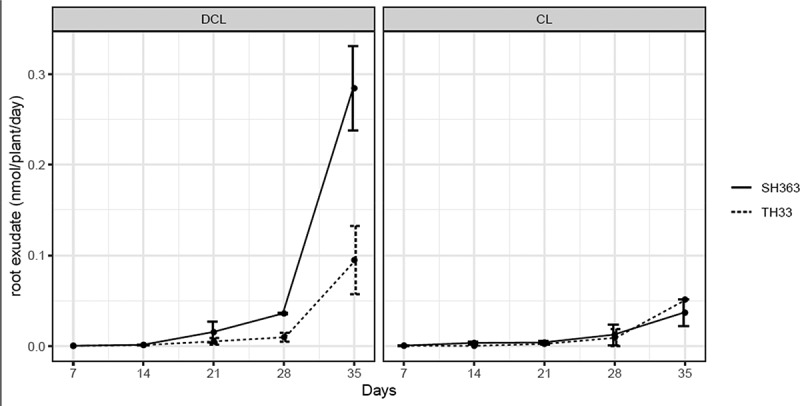
Release rate of DCL and CL by different sunflower cultivars in different time point.

Both DCL and CL were identified from root exudates of two sunflower cultivars. The quantity of DCL and CL was found varied depending on sunflower growth stages. In the first 3 weeks (7DAT and 21DAT), there was no significant difference between susceptible (SH363) and resistant (TH33) cultivar for releasing DCL. The amount of DCL gradually increased over time with a strong increase at 35 DAT in SH363 (Figure 2). The amount of CL showed no significant different difference between susceptible (SH363) and resistant cultivar (TH33). Resistant sunflower TH33 showed a similar trend as susceptible sunflower SH363; however, total amounts were lower. The DCL content rose up to 3.7 times in SH363 compared to TH33 at 28 DAT. On average, SH363 have significantly (p ≤ .05) greater DCL than TH33 after 21 DAT stages. The amount of CL showed no significant difference between susceptible (SH363) and resistant cultivar (TH33).

### 4.3 Quantity of DCL and CL in root extracts

**Figure 3.**
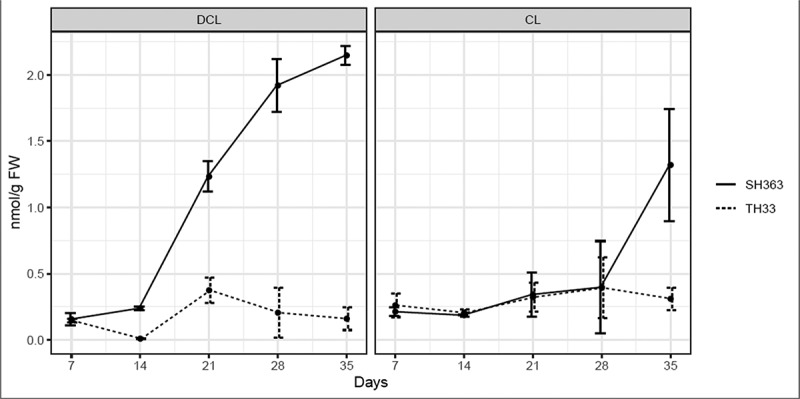
Contents of DCL and CL in root extracts of different sunflower cultivars.

Based on the results of UPLC-MS, both DCL and CL were found in the root extracts of both sunflower cultivars. The dynamic of DCL and CL showed similar patterns as root exudates which varied with sunflower growth stages and cultivars (Figure 3). In general, the total amount of DCL in root extracts of susceptible cultivar was higher than that of resistant cultivar at the same time points tested except at 7 DAT. The content of DCL in SH363 root extracts was lower at early stage (7, 14 DAT), then increased rapidly over time approaching the highest quantity at 35 DAT in the range of 0.16–2.15 nmol/g. The amount of DCL in TH33 root extracts varied with growth stages, however it was lower than SH363 at the same time point in the range of 0.01–0.37 nmol/g. SH363 showed at 35 DAT higher production of DCL in root extracts that rose up to 14 times compared to TH33. In contrast to the DCL variation, CL did not vary significantly (p ≤ .05) among the two cultivars before 28 DAT. However, SH363 provided higher content of CL in root extracts at 35 DAT compared to TH33. In TH33, the quantity of DCL and CL was lower during 7 DAT to 28 DAT, followed by a decrease at 35 DAT reach the highest content difference with SH363.

### 4.4 *Effect of root exudates on germination of* O. cumana

**Figure 4.**
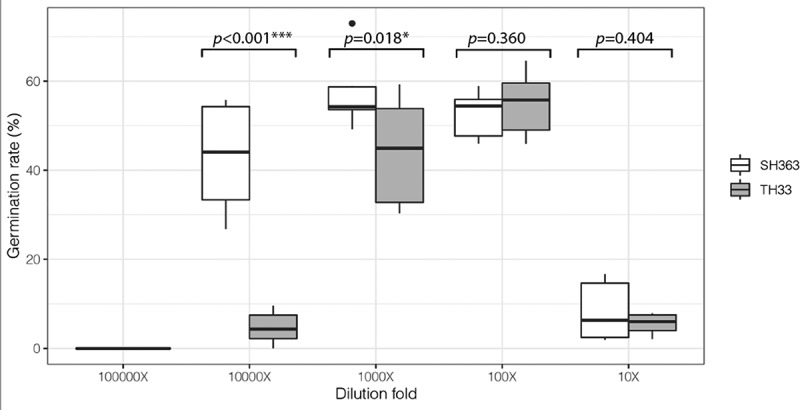
Effects of sunflower root exudates on germination (%) of *O. cumana*. Final concentrations of root exudates were diluted from crude root exudate. Welch Two Sample t-test was used the test whether the root exudates have significantly affect of germination (%) of *O. cumana* under the different concentrations.

Based on above results, different content of DCL in root exudates of SH363 and TH33 suggested that effects of root exudates from these two cultivars on *O. cumana* germination may be different. To understand this, the root exudates of susceptible and resistant sunflower cultivars collected at 28 DAT were tested (Figure 4). Different germination rates of *O. cumana* seeds were observed when treated with root exudates of SH363 and TH33 with 10,000× dilutions (crude root exudate). SH363 showed higher germination (43.14%) compared to TH33 (5.52%) when root exudates were diluted to 10,000×. The germination rate of *O. cumana* with root exudates dilution of susceptible cultivar was 8.5 times higher of the resistant cultivar TH33.

## 5 Discussion

Germination of *O. cumana* is the first stage of its life history and trigged by germination stimulants of host plant before attachment. Among germination stimulants isolated from sunflower, DCL and CL were considered as germination and gemtube inducer on *O. cumana*.^25,26^ Since DCL and CL exudation are linked in germination and gemtube of *O. cumana*, we focused on these two compounds in different sunflower cultivars and the dynamic with the growth stages of sunflower. Based on our results, both DCL and CL were detected in both root exudates and extracts of these two sunflower cultivars. However, the quantity of DCL was significantly higher in the SH363 root exudates than TH33 after 21 DAT. This indicated that the different effects of root exudates on *O. cumana* may be existed. Further experiment showed the root exudate of susceptible sunflower provided significantly higher germination rates compared with the resistant cultivar at the 10,000-fold diluted concentration. The results of root exudate activity agree with the DCL content in different cultivars. The dynamic of DCL in root exudate showed a similar pattern like in root extracts of these two sunflower cultivars during the development stages. Higher DCL/CL inside sunflower root also leads to more exudation of the products. Otherwise, the results are in agreement with those of ^13^ who considered the lower stimulants of broomrape germination were responsible for resistance to *O. cumana*. Based on our result, lower quantity of DCL in root exudates of resistant sunflower might partly explain the resistance of sunflower TH33 to *O. cumana*.

In addition, it was found DCL was detected at a higher quantity in root and root exudation than CL at the same time point. This finding is different with previous report ^23^ that DCL was negligible under any aquaculture conditions. It is suggested that different sunflower cultivars or nutritional condition are responsible for germination stimulants difference.

Based on our results, germination stimulants are released throughout the growth period of sunflower and varied in different sunflower cultivars. As described previously, different host plants release different mixtures of stimulants.^28,29^ It mainly plays a role in the early induction of *O. cumana* seed germination and gemtube.^26^ Moreover, DCL and CL were found increasing with developmental stage, which indicates that these compounds might have other physiological activities.

In our study, we collected samples using hydroponic culture which might differ considerably from real soil environmental. Sufficient concentration of stimulant in the soil is critical for broomrape germination at early stage. In addition, the coexistence of different stimulant compounds in the root exudate should be further considered. Further research will be performed through physiological activity and ecology role of DCL to explain the result.

## 6 Conclusions

In this study, we investigate specific stimulant compounds, DCL and CL, in root and root exudate of resistant and susceptible sunflower cultivars. We found that the quantity of DCL in root exudate and root extract of sunflower was increasing with the growth stage of sunflower. In addition, the results showed that higher amount of DCL present in root and root exudate of susceptible cultivar than in resistant cultivar.
